# Supplementary material for: The Systems Biology Research Tool: evolvable open-source software
Source: BMC Syst Biol. 2008 Jun 29;2:55. doi: 10.1186/1752-0509-2-55 (PMC2446383; doi:10.1186/1752-0509-2-55)
Supplement: Additional file 1 — SBRT Archive. An archive of the current version of the Systems Biology Research Tool. [file 1752-0509-2-55-S1.zip › sbrt-1.4.0/doc/developers_guide/api/sbrt/kernel/fba/proc/ObjectiveFunctionAnalysis.html]

ObjectiveFunctionAnalysis


|  |  |  |
| --- | --- | --- |
| **Overview** \| **Package** \| **Class** \| **Use** \| **Tree** \| **Deprecated** \| **Index** \| **Help** | |  |
| **PREV CLASS**   **NEXT CLASS** | **FRAMES**    **NO FRAMES**     **All Classes** |  |
| SUMMARY: NESTED \| FIELD \| CONSTR \| METHOD | DETAIL: FIELD \| CONSTR \| METHOD |  |


---


## sbrt.kernel.fba.proc Class ObjectiveFunctionAnalysis

```
java.lang.Object
  sbrt.kernel.fba.proc.FbaProcess<FbaOptComputer.Results>
      sbrt.kernel.fba.proc.ObjectiveFunctionAnalysis
```

**All Implemented Interfaces:**: KernelProcess<FbaOptComputer.Results>, MonitoredProcess<FbaOptComputer.Results>

---

``` public final class ObjectiveFunctionAnalysis extends FbaProcess<FbaOptComputer.Results> implements MonitoredProcess<FbaOptComputer.Results> ```

This class is used to perform objective function experiments.

**Author:**
:   This class was written and documented by Jeremiah Wright
    while in the Wagner lab.

---

**Constructor Summary**

`ObjectiveFunctionAnalysis(FbaOptComputer computer)`
  
          Constructs a new objective function experiment.


| **Method Summary** | |
| --- | --- |
| `Fluxome` | `getFluxome()`             Returns the fluxome used in this experiment. |
| `OptimizationSense` | `getOptimizationSense()`             Returns the current optimization sense. |
| `ProgressMonitor` | `getProgress()`             Returns a progress monitor for this experiment. |
| `void` | `run(ResultsManager<FbaOptComputer.Results> resultsManager)`             Runs this process and adds the generated results to the provided results manager via its `ResultsManager.addData(Object)` method. |
| `void` | `setObjectiveFunctionSource(java.lang.Iterable<? extends MathExpr<java.lang.String>> objectiveSource)`             Sets the objective function input source. |
| `void` | `setOptimizationSense(OptimizationSense sense)`             Sets the optimization sense to the provided value. |

**Methods inherited from class sbrt.kernel.fba.proc.FbaProcess**

`toString`

**Methods inherited from class java.lang.Object**

`clone, equals, finalize, getClass, hashCode, notify, notifyAll, wait, wait, wait`

**Constructor Detail**

### ObjectiveFunctionAnalysis

```
public ObjectiveFunctionAnalysis(FbaOptComputer computer)
```

:   Constructs a new objective function experiment.

    **Parameters:**: `computer` - the computer with which all results will be computed.


**Method Detail**

### getFluxome

```
public Fluxome getFluxome()
```

:   Returns the fluxome used in this experiment.

    :   **Specified by:**: `getFluxome` in class `FbaProcess<FbaOptComputer.Results>`
    :   **Returns:**: the fluxome used in this experiment.

---


### getOptimizationSense

```
public OptimizationSense getOptimizationSense()
```

:   Returns the current optimization sense.

    :   **Returns:**: the current optimization sense.

---


### setOptimizationSense

```
public void setOptimizationSense(OptimizationSense sense)
```

:   Sets the optimization sense to the provided value.

    :   **Parameters:**: `sense` - an optimization sense.

---


### setObjectiveFunctionSource

```
public void setObjectiveFunctionSource(java.lang.Iterable<? extends MathExpr<java.lang.String>> objectiveSource)
```

:   Sets the objective function input source.

    :   **Parameters:**: `objectiveSource` - a source of objective functions whose optimal values are to be computed.

---


### getProgress

```
public ProgressMonitor getProgress()
```

:   Returns a progress monitor for this experiment.

    :   **Specified by:**: `getProgress` in interface `MonitoredProcess<FbaOptComputer.Results>`
    :   **Returns:**: a progress monitor for this experiment.

---


### run

```
public void run(ResultsManager<FbaOptComputer.Results> resultsManager)
```

:   Runs this process and adds the generated
    results to the provided results manager via
    its `ResultsManager.addData(Object)`
    method.

    An objective function is retrieved from the objective source, its
    optimal value is computed, and the results are added to the
    provided results manager. This is repeated until the end of the objective
    source is reached.

    :   **Specified by:**: `run` in interface `KernelProcess<FbaOptComputer.Results>`
    :   **Parameters:**: `resultsManager` - the results manager to which the results will be added.


---


|  |  |  |
| --- | --- | --- |
| **Overview** \| **Package** \| **Class** \| **Use** \| **Tree** \| **Deprecated** \| **Index** \| **Help** | |  |
| **PREV CLASS**   **NEXT CLASS** | **FRAMES**    **NO FRAMES**     **All Classes** |  |
| SUMMARY: NESTED \| FIELD \| CONSTR \| METHOD | DETAIL: FIELD \| CONSTR \| METHOD |  |


---
